# Assessment of Unmet Health-Related Social Needs Among Patients With Mental Illness Enrolled in Medicare Advantage

**DOI:** 10.1001/jamanetworkopen.2022.39855

**Published:** 2022-11-02

**Authors:** Omolola E. Adepoju, Winston Liaw, Nick C. Patel, Jeremiah Rastegar, Matthew Ruble, Stephanie Franklin, Andrew Renda, Ezemenari Obasi, LeChauncy Woodard

**Affiliations:** 1 Tillman J. Fertitta Family College of Medicine, University of Houston, Houston, Texas; 2 Humana Integrated Health System Sciences Institute, Houston, Texas; 3 Humana Inc, Louisville, Kentucky; 4 Department of Psychological, Health and Learning Sciences, College of Education, University of Houston, Houston, Texas; 5 HEALTH Research Institute, University of Houston, Houston, Texas

## Abstract

**Importance:**

A large body of literature has found associations between unmet health-related social needs (HRSNs) and adverse mental health outcomes. A comparative analysis of the risks associated with HRSNs among patients with varying severity of mental illness and an assessment of how these risks compare with those of individuals without mental illness are needed.

**Objective:**

To examine the prevalence and risks of HRSNs among patients with serious and persistent mental illness (SPMI), patients with mental health diagnoses but no serious and persistent mental illness (non-SPMI), and patients with both SPMI and non-SPMI compared with individuals without mental illness.

**Design, Setting, and Participants:**

This retrospective cohort study used data from the Accountable Health Communities HRSN Screening Tool surveys, which target a nationally representative sample of Medicare Advantage members of a large payer (Humana Inc). The surveys were conducted between October 16, 2019, and February 29, 2020. Of the initial 329 008 eligible Medicare Advantage enrollees, 70 273 responded to the survey (21.4% response rate). Of those, 56 081 respondents (79.8%) had complete survey responses and were included in the final analytic sample.

**Main Outcomes and Measures:**

Outcomes of interest included 7 HRSNs (financial strain, food insecurity, housing instability, housing quality, severe loneliness, transportation problems, and utility affordability) based on responses to the survey. The major independent variable was the presence of mental illness up to 12 months preceding the date of survey completion. Codes indicating mental illness listed as the primary, principal, or secondary diagnoses of a patient's inpatient or outpatient medical claims data were identified, and participants were grouped into 4 cohorts: SPMI, non-SPMI, SPMI plus non-SPMI, and no mental illness.

**Results:**

Among 56 081 older adults, the mean (SD) age was 71.31 (8.59) years; 32 717 participants (58.3%) were female, and 43 498 (77.6%) were White. A total of 21 644 participants (38.6%) had at least 1 mental illness diagnosis in the past year, 30 262 (54.0%) had an HRSN, and 14 163 (25.3%) had both mental illness and an HRSN. Across all specific HRSNs, the odds of experiencing the respective HRSN was most substantial for those with SPMI plus non-SPMI vs those with only non-SPMI or SPMI. The HRSN with the largest risk differences among the study cohorts was severe loneliness; compared with the cohort without mental illness, the non-SPMI cohort had 2.07 times higher odds (95% CI, 1.84-2.32; *P* < .001), the SPMI cohort had 3.35 times higher odds (95% CI, 3.03-3.71; *P* < .001), and the SPMI plus non-SPMI cohort had 5.13 times higher odds (95% CI, 4.68-5.61; *P* < .001) of severe loneliness.

**Conclusions and Relevance:**

In this study, the increased risk of having HRSNs associated with SPMI, alone or in combination with non-SPMI, emphasizes the need for more targeted interventions to address social needs in this vulnerable population.

## Key Points

**Question:**

What are the prevalence and risks associated with health-related social needs (HRSNs) among individuals with and without serious and persistent mental illness (SPMI)?

**Findings:**

In this cohort study of 56 081 Medicare Advantage enrollees with and without mental illness, 38.6% had at least 1 mental illness diagnosis, 54.0% had an HRSN, and 25.3% had both mental illness and an HRSN in the past year. The association of mental illness with the presence of HRSNs was most substantial among those with both SPMI and non-SPMI than those with only SPMI or non-SPMI.

**Meaning:**

These findings suggest that basic social needs, such as safe housing and food security, remain challenging in one of the most vulnerable populations.

## Teaser

This cohort study uses data from the Accountable Health Communities Health-Related Social Needs Screening Tool surveys to assess the association between mental illness and health-related social needs among Medicare Advantage enrollees.

## Introduction

Unmet health-related social needs (HRSNs, or the social risk factors that individuals want to address)^1^ have been associated with inequitable health outcomes that are largely avoidable and remediable. A large body of literature has found an association between unmet HRSNs and undesirable health outcomes,^2^ leading to calls for health care organizations to develop and implement standardized approaches to systematically assess and address HRSNs.^3^ Since the development of the Centers for Medicare and Medicaid Services (CMS) Accountable Health Communities (AHC) HRSN Screening Tool,^4^ increased attention has been paid to identifying and documenting the prevalence of HRSNs among populations served by the CMS. Additional tools to assess HRSNs include the Protocol for Responding to and Assessing Patient Assets, Risks, and Experiences (PRAPARE) developed by the National Association of Community Health Centers and partners^5^ and the Senior-Specific Social Needs Screener developed by West Health.^6^ Although these screening tools exist, many clinicians are unaware of how to best to use these screening tools to connect their patients to resources that address social needs.^7,8,9^ To do so, new pathways need to be created across the historical silos of public health, primary care, and mental health.^10,11^

The need to address mental health is serious in the US, where 1 in 5 adults experiences mental illness each year and 11.2 million adults live with a serious mental illness.^12^ Compared with the general population, those with mental illness have higher morbidity and mortality, and those with serious mental illness die a decade earlier.^13,14,15,16^ These premature deaths reflect not only higher rates of chronic diseases, such as diabetes, cancer, and cardiovascular disease, but unmet social needs that emerge from stigma, isolation, and homelessness.^17,18^ Increasing mental illness–related deaths nationwide suggest that the number of individuals in the US concurrently dealing with social and mental problems has increased—and likely will continue to increase—unless new strategies are implemented to simultaneously address social needs, physical health, and mental health.^19^ This decrease in life expectancy is particularly concerning because individuals with serious mental illness have distinct challenges that make treatment engagement more difficult in this patient population.^20^

Although many individuals in the US have HRSNs, those with mental illness are at particularly high risk.^21,22^ For example, mental illness has been found to have implications for financial insecurity, both directly and indirectly. Compared with individuals with no, mild, or moderate disease, those with serious mental illness have lower educational attainment, employment rates, and salaries when employed, leading to higher rates of poverty.^23,24^ Unemployed individuals with serious mental illness are more likely to require hospitalization, which can drain financial resources and exacerbate debt.^25,26^ With limited resources, individuals with mental illness often lack adequate food and stable housing,^27,28^ making it difficult to manage their diseases.^29,30^ Furthermore, they generally reside in neighborhoods without safe spaces to exercise and access to healthy food options.^17,31^ In addition to these stressors, the stigma of having mental illness reduces opportunities to engage with others, leading to loneliness rates twice that of the general population.^15^ Having 1 unmet social need can trigger a downward spiral in which other social needs emerge, health care and medications are inaccessible, and mental health further degrades.^32^ The converse is also true, with poverty and violence potentially leading to mental health issues,^33^ making it difficult to assess whether unmet social needs are associated with the onset of mental illness or vice versa and highlighting the value of approaches that address both factors.^34^

The importance of addressing this intersection of health and HRSNs is greater with the transition to value-based care, which provides incentives for health care organizations to address upstream factors^35^ that impact health outcomes. This issue is particularly true for Medicare Advantage plans, which are responsible for the total cost of care in older adult populations. Because individuals may have multiple social needs, a comparative analysis of the risks associated with different HRSNs among patients with mental illness and assessment of how these risks compare with those of individuals without mental illness are needed. To our knowledge, few studies have fully examined this association; studies have instead focused on smaller subpopulations, such as those with single social needs, those with individual mental health diagnoses, or those from specific racial and/or ethnic groups. In addition, there is a lack of research on the severity of mental illness and the risk of multiple social needs, which is an important area of research to improve quality of care and reduce costs in this population.

The objective of this cohort study was to examine the prevalence and risks of 7 HRSNs among patients with serious and persistent mental illness (SPMI), patients with mental health diagnoses but no serious and persistent mental illness (non-SPMI), and patients with both SPMI and non-SPMI compared with individuals without mental illness. By focusing on a spectrum of HRSN domains, this study allows for a better understanding of the additive potential of HRSNs, which may be useful for risk stratification and disease management. The study’s findings may also provide evidence to facilitate the advancement of policies that promote the integration of physical, mental, and social health.

## Methods

### Study Sample and Data Sources

This cohort study was approved by an independent institutional review board in May 2020, and a waiver of informed consent granted because of the use of deidentified data. Data on participant HRSNs were obtained from the AHC HRSN Screening Tool surveys^4^ included in administrative data from Humana Inc. The administrative claims database also includes enrollment files, medical claims, and outpatient pharmacy claims data from Humana Inc, a US-based company that provides Medicare Advantage, stand-alone Medicare prescription drug plans, and commercial plan offerings. The AHC HRSN Screening Tool surveys were conducted between October 16, 2019, and February 29, 2020, via interactive voice response call, short messaging service text, and/or email, depending on contact information availability.^4^ Surveys included questions about 7 HRSNs: financial strain, food insecurity, housing instability, housing quality, severe loneliness, transportation problems, and utility affordability.

For the purposes of this study, individuals were included if they (1) were age 18 to 89 years; (2) were enrolled in Medicare Advantage, including those with disabled status, special needs plans (SNPs), and dual eligibility; (3) were continuously enrolled for 12 months before the survey date (ie, index date); and (4) fully completed (with no missing values) the AHC HRSN Screening Tool survey. Individuals were excluded if they were enrolled in group plans such as commercial administrative services–only groups that are contractually excluded from research. Of the initial 329 008 eligible Medicare Advantage enrollees, 70 273 responded to the survey (21.4% response rate). The demographic characteristics of survey respondents vs nonrespondents and the standardized mean differences (SMDs) between both groups are shown in Table 1. Of the respondents, 56 081 (79.8%) had complete survey responses, constituting the final analytic sample.

**Table 1.** Demographic Characteristics of Survey Respondents vs Nonrespondents

Characteristic	Individuals, No. (%)			SMD
	Total (N = 329 008)	Responded to survey		
		Yes (n = 70 273)	No (n = 258 735)	
Age, mean (SD), y	70.80 (9.66)	71.39 (8.66)	70.64 (9.91)	0.080
Age category, y				
18-34	1400 (0.4)	112 (0.2)	1288 (0.5)	0.059
35-49	9831 (3.0)	1194 (1.7)	8637 (3.3)	0.105
50-64	51 115 (15.5)	10 505 (14.9)	40 610 (15.7)	0.021
65-79	210 744 (64.1)	46 836 (66.6)	163 908 (63.3)	0.069
80-89	55 918 (17.0)	11 626 (16.5)	44 292 (17.1)	0.015
Sex				
Female	181 354 (55.1)	40 733 (58.0)	140 621 (54.3)	0.073
Male	147 654 (44.9)	29 540 (42.0)	118 114 (45.7)	0.060
Race				
Black	56 004 (17.0)	12 580 (17.9)	43 424 (16.8)	0.030
White	251 898 (76.6)	54 158 (77.1)	197 740 (76.4)	0.015
Other^a^	17 237 (5.2)	2739 (3.9)	14 498 (5.6)	0.080
Unknown	3869 (1.2)	796 (1.1)	3073 (1.2)	0.005
Population density				
Rural	37 488 (11.4)	7784 (11.1)	29 704 (11.5)	0.013
Suburban	82 583 (25.1)	17 700 (25.2)	64 883 (25.1)	0.003
Urban	201 993 (61.4)	43 246 (61.5)	158 747 (61.4)	0.004
Unknown	6944 (2.1)	1543 (2.2)	5401 (2.1)	0.007
Region				
Midwest	70 876 (21.5)	15 293 (21.8)	55 583 (21.5)	0.007
Northeast	10 409 (3.2)	2040 (2.9)	8369 (3.2)	0.019
South	206 517 (62.8)	44 156 (62.8)	162 361 (62.8)	0.002
West	41 206 (12.5)	8784 (12.5)	32 422 (12.5)	0.001
Dual eligibility	89 819 (27.3)	18 535 (26.4)	71 284 (27.6)	0.026
Special needs plan	19 630 (6.0)	4590 (6.5)	15 040 (5.8)	0.030
Low-income subsidy	66 735 (20.3)	13 688 (19.5)	53 047 (20.5)	0.026
SES index score, mean (SD)^b^	51.84 (7.22)	51.90 (6.98)	51.82 (7.28)	0.011

Abbreviations: SES, socioeconomic status; SMD, standardized mean difference.

^a^ Other races include American Indian, Asian, and 2 or more races.

^b^ Score range, 0 to 100, with higher scores indicating higher socioeconomic levels.

The AHC HRSN Screening Tool data were merged with enrollment and medical claims data. Enrollment data contained demographic characteristics, including date of birth, sex, race and ethnicity, and start and end dates of plan coverage. Medical claims data, which included diagnostic codes from the *International Classification of Diseases, Tenth Revision, Clinical Modification* (*ICD-10-CM*), were used to identify patients diagnosed with mental illness, as defined by the *Diagnostic and Statistical Manual of Mental Disorders* (Fifth Edition).^36^

### Study Outcomes and Variables

Each of the 7 HRSNs represented an outcome of interest and included (1) financial strain, (2) food insecurity, (3) housing instability, (4) housing quality, (5) severe loneliness, (6) transportation problems, and (7) utility affordability. Prevalence for each HRSN was assessed based on responses to the survey questions. Survey responses were treated as dichotomous (positive or negative screening result). We also examined the incremental consequences of HRSNs based on the total number of HRSNs reported.

**Figure 1.**
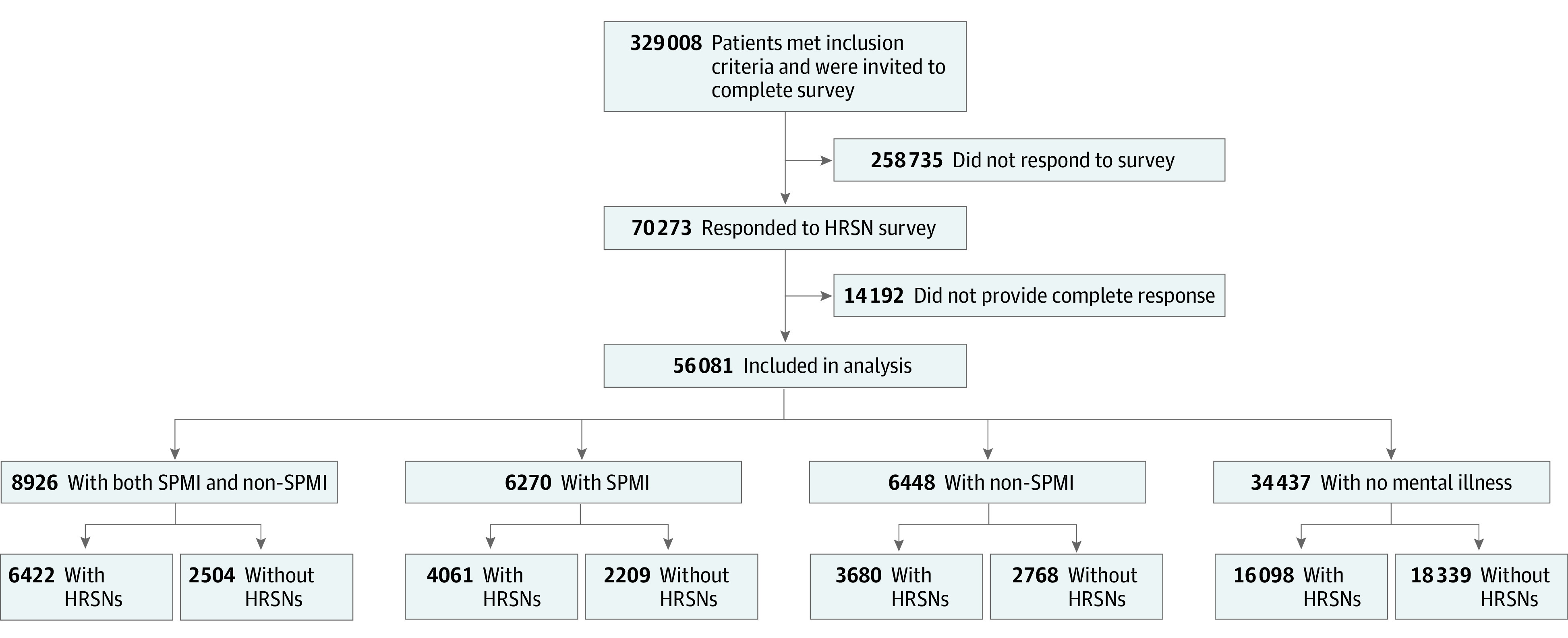
Cohort Diagram HRSN indicates health-related social need; and SPMI, serious persistent mental illness.

The major independent variable for the adjusted logistic regression models was the presence of mental illness up to 12 months before the date of survey completion. We identified codes indicating mental illness listed as the primary, principal, or secondary diagnoses of a patient's inpatient or outpatient medical claims data, and participants were grouped into 4 study cohorts (SPMI, non-SPMI, SPMI plus non-SPMI, and no mental illness) following the criteria outlined in a Milliman research report^37^ on the economic the impact of integrated medical-behavioral health care and consistent with definitions used by other researchers (Figure 1).^38,39,40^ Accordingly, SPMI was defined as having any of the following diagnoses: bipolar disorders (*ICD-10-CM* codes F30.XX, F31.XX, and F34.0), major depressive disorders (*ICD-10-CM* codes F32.0-F32.5, F32.89, F32.9, F33.XX, and F34.1), or schizophrenia and other psychotic disorders (*ICD-10-CM* codes F20.XX, F21, F22, F24, F25, F28, and F29). Non-SPMI was defined as having any the following diagnoses: adjustment disorders (*ICD-10-CM* codes F43.0, F43.2X, F43.8, and F43.9), anxiety disorders (*ICD-10-CM* codes F40.XXX, F41.X, and F42.X), dissociative and conversion disorders (*ICD-10-CM* code F44.XX), eating disorders (*ICD-10-CM* code F50.XX), neurocognitive disorders (*ICD-10-CM* codes F01.XX, F02.XX, F03.XX, and G30.X), other mood disorders (*ICD-10-CM* codes F34.8X, F34.9, and F39), posttraumatic stress disorder (*ICD-10-CM* code F43.1X), or somatoform disorders (*ICD-10-CM* code F45.XX). Patients who had both SPMI and non-SPMI diagnoses were included in the SPMI plus non-SPMI cohort. All 3 cohorts (SPMI, non-SPMI, and SPMI plus non-SPMI) were mutually exclusive, allowing us to examine the association of different mental illnesses with HRSNs.

Covariates that were adjusted for in the logistic regression models included the participant’s age on the index date, sex, geographic region based on the participant’s state of residence on the index date (Midwest, Northeast, South, and West); rural vs urban status; race and ethnicity (Black, White, and other [including American Indian, Asian, and ≥2 races; these categories were combined owing to small sample sizes and inaccuracies in CMS classification for non-Black and non-White beneficieries], and unknown); socioeconomic status index score (range, 0-100, with higher scores indicating higher socioeconomic levels) to summarize area-level information on socioeconomic status incorporating occupation, income, educational level, and housing; dual eligibility for Medicare and Medicaid; enrollment in an SNP; low-income subsidy status (Medicare beneficiaries with incomes <150% of the poverty limit); presence of alcohol-related disorders *(ICD-10-CM* code F10.XXX); presence of drug-related disorders (*ICD-10-CM* codes F.11.XXX-F19.XXX); Elixhauser Comorbidity Index score; preindex all-cause health care resource use (inpatient hospitalizations, emergency department visits, and outpatient visits); and preindex total out-of-pocket expenses. The Elixhauser Comorbidity Index estimates the risk of death and uses diagnostic codes to capture disease burden.^41^ Incorporating this index was important because these comorbidities have potential implications for mental illness and HRSNs.

### Statistical Analysis

Descriptive analyses measuring frequencies, proportions, means, and SDs were used to describe participant demographic and clinical characteristics for the study cohorts. For each HRSN domain, cross-tabulations were used to examine the relative prevalence within the cohorts with mental illness. Seven logistic regression models were used to assess the association between mental illness in the SPMI, non-SPMI, and SPMI plus non-SPMI cohorts and the presence of individual HRSNs, adjusting for demographic and clinical characteristics and other covariates. For all of these models, the cohort without mental illness served as the reference category. All data management and analyses were performed using SAS software, version 9.4 (SAS Institute Inc). All statistical tests were 2-sided, and findings were considered statistically significant at *P* < .05.

## Results

### Demographic and Clinical Characteristics

The demographic characteristics of survey respondents (n = 70 273) vs nonrespondents (n = 258 735) and the associated SMDs are shown in Table 1. All SMDs were less than 0.100, revealing minimal differences between respondents and nonrespondents. For example, among respondents vs nonrespondents, the mean (SD) age was 71.39 (8.66) years vs 70.64 (9.91) years (SMD, 0.080), and most were female (40 733 individuals [58.0%] vs 140 621 individuals [54.3%]; SMD, 0.073) and White (54 158 individuals [77.1%] vs 197 740 individuals [76.4%]; SMD, 0.015).

The demographic characteristics of the study population, stratified by the 4 study cohorts, are shown in Table 2. Among 56 081 participants, the mean (SD) age was 71.31 (8.59) years; 32 717 participants (58.3%) were female, 23 364 (41.7%) were male, 9937 (17.7%) were Black, 43 498 (77.6%) were White, 2014 (3.6%) were of other races (including American Indian, Asian, and ≥2 races), and 632 (1.1%) were of unknown race. Most participants resided in southern states (35 036 [62.5%]) and urban areas (34 397 [61.3%]). A total of 6448 participants (11.5%) were in the non-SPMI cohort, 6270 (11.2%) were in the SPMI cohort, 8926 (15.9%) were in the SPMI plus non-SPMI cohort, and 34 437 (61.4%) were in the cohort without mental illness.

**Table 2.** Demographic and Clinical Characteristics of Study Cohorts

Characteristic	Participants, No. (%)				
	Total (N = 56 081)	No mental illness (n = 34 437)	Non-SPMI (n = 6448)	SPMI (n = 6270)	SPMI plus non-SPMI (n = 8926)
**Demographic**					
Age, mean (SD), y	71.31 (8.59)	75.53 (7.63)	72.01 (8.86)	69.39 (9.30)	67.47 (10.09)
Age category, y					
18-34	86 (0.2)	37 (0.1)	7 (0.1)	11 (0.2)	31 (0.3)
35-49	924 (1.6)	267 (0.8)	98 (1.5)	154 (2.5)	405 (4.5)
50-64	8466 (15.1)	3286 (9.5)	985 (15.3)	1445 (23.0)	2750 (30.8)
65-79	37 596 (67.0)	24 871 (72.2)	4049 (62.8)	3909 (62.3)	4767 (53.4)
80-89	9009 (16.1)	5976 (17.4)	1309 (20.3)	751 (12.0)	973 (10.9)
Sex					
Female	32 717 (58.3)	17 880 (51.9)	4227 (65.6)	4202 (67.0)	6408 (71.8)
Male	23 364 (41.7)	16 557 (48.1)	2221 (34.4)	2068 (33.0)	2518 (28.2)
Geographic region					
Midwest	12 414 (22.1)	7785 (22.6)	1401 (21.7)	1278 (20.4)	1950 (21.8)
Northeast	1616 (2.9)	1032 (3.0)	181 (2.8)	134 (2.1)	269 (3.0)
South	35 036 (62.5)	21 162 (61.5)	4115 (63.8)	4025 (64.2)	5734 (64.2)
West	7015 (12.5)	4458 (12.9)	751 (11.6)	833 (13.3)	973 (10.9)
Population density					
Rural	6308 (11.2)	3903 (11.3)	786 (12.2)	617 (9.8)	1002 (11.2)
Suburban	14 136 (25.2)	8742 (25.4)	1649 (25.6)	1449 (23.1)	2296 (25.7)
Urban	34 397 (61.3)	21 058 (61.1)	3848 (59.7)	4056 (64.7)	5435 (60.9)
Unknown	1240 (2.2)	734 (2.1)	165 (2.6)	148 (2.4)	193 (2.2)
Race					
Black	9937 (17.7)	6840 (19.9)	941 (14.6)	1030 (16.4)	1126 (12.6)
White	43 498 (77.6)	25 768 (74.8)	5280 (81.9)	4957 (79.1)	7493 (83.9)
Other^a^	2014 (3.6)	1361 (4.0)	174 (2.7)	227 (3.6)	252 (2.8)
Unknown	632 (1.1)	468 (1.4)	53 (0.8)	56 (0.9)	55 (0.6)
Dual eligibility	14 577 (26.0)	7093 (20.6)	1693 (26.3)	2101 (33.5)	3690 (41.3)
Special needs plan	3595 (6.4)	1679 (4.9)	379 (5.9)	597 (9.5)	940 (10.5)
Low-income subsidy	10 726 (19.1)	5121 (14.9)	1231 (19.1)	1589 (25.3)	2785 (31.2)
SES index score, mean (SD)^b^	51.91 (6.91)	51.93 (6.98)	51.87 (7.01)	51.85 (6.98)	51.92 (6.50)
**Clinical**					
Unique neuropsychiatric diagnoses, mean (SD)	0.63 (0.94)	NA	1.11 (0.35)	1.04 (0.22)	2.40 (0.71)
SPMI diagnostic category					
Bipolar and related disorders	1655 (3.0)	NA	NA	1453 (23.2)	1202 (13.5)
Major depressive disorder	14 328 (25.5)	NA	NA	5889 (93.9)	8439 (94.5)
Schizophrenia spectrum disorders and other psychotic disorders	673 (1.2)	NA	NA	210 (3.3)	463 (5.2)
Non-SPMI diagnostic category					
Adjustment disorder	1977 (3.5)	NA	859 (13.3)	NA	1118 (12.5)
Alcohol-related disorder	3794 (6.8)	1385 (4.0)	425 (6.6)	653 (10.4)	1331 (14.9)
Anxiety disorder	12 649 (22.6)	NA	4749 (73.7)	NA	7900 (88.5)
Dissociative and conversion disorder	119 (0.2)	NA	27 (0.4)	NA	92 (1.0)
Drug-related disorder	8985 (16.0)	3897 (11.3)	1160 (18.0)	1338 (21.3)	2590 (29.0)
Eating disorder	94 (0.2)	NA	34 (0.5)	NA	60 (0.7)
Neurocognitive disorder	1677 (3.0)	NA	862 (13.4)	NA	815 (9.1)
Other mood disorder	969 (1.7)	NA	398 (6.2)	NA	571 (6.4)
PTSD	771 (1.4)	NA	140 (2.2)	NA	631 (7.1)
Somatoform disorder	277 (0.5)	NA	111 (1.7)	NA	166 (1.9)
Elixhauser Comorbidity Index score, mean (SD)	3.04 (2.63)	2.55 (2.38)	3.11 (2.53)	3.90 (2.77)	4.31 (2.89)

Abbreviations: NA, not applicable; PTSD, posttraumatic stress disorder; SES, socioeconomic status; SPMI, serious persistent mental illness.

^a^ Other races include American Indian, Asian, and 2 or more races.

^b^ Score range, 0 to 100, with higher scores indicating higher socioeconomic levels.

Compared with the overall study population, female and White patients were overrepresented in the non-SPMI cohort (4227 [65.6%] female and 5280 [81.9%] White participants), the SPMI cohort (4202 [67.0%] female and 4957 [79.1%] White participants), and the SPMI plus non-SPMI cohort (6408 [71.8%] female and 7493 [83.9%] White participants). In contrast, Black individuals were overrepresented in the cohort without mental illness (6840 participants [19.9%]). Individuals with dual eligibility for Medicare and Medicaid were more likely to be in the 3 cohorts with mental illness (non-SPMI: 1693 participants [26.3%]; SPMI: 2101 participants [33.5%]; SPMI plus non-SPMI: 3690 participants [41.3%]) than in the cohort without mental illness (7093 participants [20.6%]). Similar patterns were observed regarding enrollment in an SNP (non-SPMI: 379 participants [5.9%]; SPMI: 597 participants [9.5%]; SPMI plus non-SPMI: 940 participants [10.5%]; no mental illness: 1679 participants [4.9%]) and low-income subsidy status (non-SPMI: 1231 participants [19.1%]; SPMI: 1589 participants [25.3%]; SPMI plus non-SPMI: 2785 participants [31.2%]; no mental illness: 5121 participants [14.9%]).

Overall, 21 644 participants (38.6%) had at least 1 mental illness diagnosis in the 12 months before their survey date (Table 2). The most prevalent disorders among those diagnosed with non-SPMI were anxiety disorders (4749 participants [73.7%]), and the most prevalent disorders among those diagnosed with SPMI were major depressive disorders (5889 participants [93.9%]). Within the SPMI plus non-SPMI cohort, the most common diagnoses were major depressive disorders (8439 participants [94.5%]) and anxiety disorders (7900 participants [88.5%]). Rates of alcohol-related disorders were higher in the SPMI cohort (653 participants [10.4%]) and the SPMI plus non-SPMI cohort (1331 participants [14.9%]) compared with the cohort without mental illness (1385 participants [4.0%]) and the non-SPMI cohort (425 participants [6.6%]). A total of 1453 participants (23.2%) in the SPMI cohort and 1202 participants (13.5%) in the SPMI plus non-SPMI cohort had bipolar and related disorders, and 210 participants (3.3%) in the SPMI cohort and 463 participants (5.2%) in the SPMI plus non-SPMI cohort had schizophrenia spectrum and other psychotic disorders. Drug-related disorders were more common in the non-SPMI cohort (1160 participants [18.0%]), the SPMI cohort (1338 participants [21.3%]), and the SPMI plus non-SPMI cohort (2590 participants [29.0%]) than in the cohort without mental illness (3897 participants [11.3%]). The mean (SD) Elixhauser Comorbidity Index score was 2.55 (2.38) in the cohort without mental illness, 3.11 (2.53) in the non-SPMI cohort, 3.90 (2.77) in the SPMI cohort, and 4.31 (2.89) in the SPMI plus non-SPMI cohort.

### Prevalence of Health-Related Social Needs by Mental Illness

The prevalence of incremental HRSNs by study cohort is shown in Table 3. Overall, 30 262 participants (54.0%) had an HRSN, and 14 163 (25.3%) had both mental illness and an HRSN. The proportion of individuals without an HRSN was significantly higher in the cohort without mental illness (18 339 participants [53.3%]) compared with the 3 cohorts with mental illness (non-SPMI: 2769 participants [42.9%]; SPMI: 2209 participants [35.2%]; SPMI plus non-SPMI: 2504 participants [28.1%]; *P* < .001). Although statistically significant, the proportion of individuals with 1 HRSN was similar across all cohorts (no mental illness: 7977 participants [23.2%]; non-SPMI: 1582 participants [24.5%]; SPMI: 1439 participants [23.0%]; SPMI plus non-SPMI: 1850 participants [20.7%]; *P* < .001). However, the proportion of those with 2 HRSNs was significantly higher in the cohorts with mental illness (non-SPMI: 1009 participants [15.6%]; SPMI: 1060 participants [16.9%]; SPMI plus non-SPMI: 1711 participants [19.2%]) compared with the cohort without mental illness (4185 participants [12.2%]; *P* < .001). The same pattern was observed among those with 3 HRSNs (no mental illness: 2255 participants [6.5%]; non-SPMI: 588 participants [9.1%]; SPMI: 797 participants [12.7%]; SPMI plus non-SPMI: 1256 participants [14.1%]; *P* < .001), 4 HRSNs (no mental illness: 1060 participants [3.1%]; non-SPMI: 300 participants [4.7%]; SPMI: 444 participants [7.1%]; SPMI plus non-SPMI: 833 participants [9.3%]; *P* < .001), or 5 or more HRSNs (no mental illness: 621 participants [1.8%]; non-SPMI: 201 participants [3.1%]; SPMI: 321 participants [5.1%]; SPMI plus non-SPMI: 772 participants [8.6%]; *P* < .001).

**Table 3.** Prevalence of HRSNs by Cohort

Variable	Participants, No. (%)				*P* value
	No mental illness (n = 34 437)	Non-SPMI (n = 6448)	SPMI (n = 6270)	SPMI plus non-SPMI (n = 8926)	
No. of HRSNs					
0	18 339 (53.3)	2769 (42.9)	2209 (35.2)	2504 (28.1)	<.001
1	7977 (23.2)	1582 (24.5)	1439 (23.0)	1850 (20.7)	<.001
2	4185 (12.2)	1009 (15.6)	1060 (16.9)	1711 (19.2)	<.001
3	2255 (6.5)	588 (9.1)	797 (12.7)	1256 (14.1)	<.001
4	1060 (3.1)	300 (4.7)	444 (7.1)	833 (9.3)	<.001
≥5	621 (1.8)	201 (3.1)	321 (5.1)	772 (8.6)	<.001
HRSN domain					
Financial strain	10 884 (31.6)	2663 (41.3)	3057 (48.8)	5047 (56.5)	<.001
Food insecurity	6150 (17.9)	1565 (24.3)	2008 (32.0)	3606 (40.4)	<.001
Housing instability	1815 (5.3)	448 (6.9)	580 (9.3)	1022 (11.4)	<.001
Housing quality	5691 (16.5)	1294 (20.1)	1547 (24.7)	2447 (27.4)	<.001
Severe loneliness	1084 (3.1)	446 (6.9)	766 (12.2)	1756 (19.7)	<.001
Transportation problems	2171 (6.3)	606 (9.4)	774 (12.3)	1492 (16.7)	<.001
Utility affordability	2875 (8.3)	636 (9.9)	714 (11.4)	1212 (13.6)	<.001

Abbreviations: HRSN, health-related social need; SPMI, serious persistent mental illness.

The prevalence of specific HRSNs varied significantly across the cohorts with mental illness but was generally higher in those cohorts vs the cohort without mental illness. For financial strain, the prevalence was 2663 participants (41.3%) in the non-SPMI cohort, 3057 participants (48.8%) in the SPMI cohort, and 5047 participants (56.5%) in the SPMI plus non-SPMI cohort compared with 10 884 participants (31.6%) in the cohort without mental illness (*P* < .001). For food insecurity, the prevalence was 1565 participants (24.3%) in the non-SPMI cohort, 2008 participants (32.0%) in the SPMI cohort, and 3606 participants (40.4%) in the SPMI plus non-SPMI cohort compared with 6150 participants (17.9%) in the cohort without mental illness (*P* < .001). For housing instability, the prevalence was 448 participants (6.9%) in the non-SPMI cohort, 580 participants (9.3%) in the SPMI cohort, and 1022 participants (11.4%) in the SPMI plus non-SPMI cohort compared with 1815 participants (5.3%) in the cohort without mental illness (*P* < .001). For housing quality, the prevalence was 1294 participants (20.1%) in the non-SPMI cohort, 1547 participants (24.7%) in the SPMI cohort, and 2447 participants (27.4%) in the SPMI plus non-SPMI cohort compared with 5691 participants (16.5%) in the cohort without mental illness (*P* < .001). For severe loneliness, the prevalence was 446 participants (6.9%) in the non-SPMI cohort, 766 participants (12.2%) in the SPMI cohort, and 1756 participants (19.7%) in the SPMI plus non-SPMI cohort compared with 1084 participants (3.1%) in the cohort without mental illness (*P* < .001). For transportation problems, the prevalence was 606 participants (9.4%) in the non-SPMI cohort, 774 participants (12.3%) in the SPMI cohort, and 1492 participants (16.7%) in the SPMI plus non-SPMI cohort compared with 2171 participants (6.3%) in the cohort without mental illness (*P* < .001). For utility affordability, the prevalence was 636 participants (9.9%) in the non-SPMI cohort, 714 participants (11.4%) in the SPMI cohort, and 1212 participants (13.6%) in the SPMI plus non-SPMI cohort compared with 2875 participants (8.3%) in the cohort without mental illness (*P* < .001).

### Adjusted Models of the Association Between Mental Illness and Health-Related Social Needs

**Figure 2.**
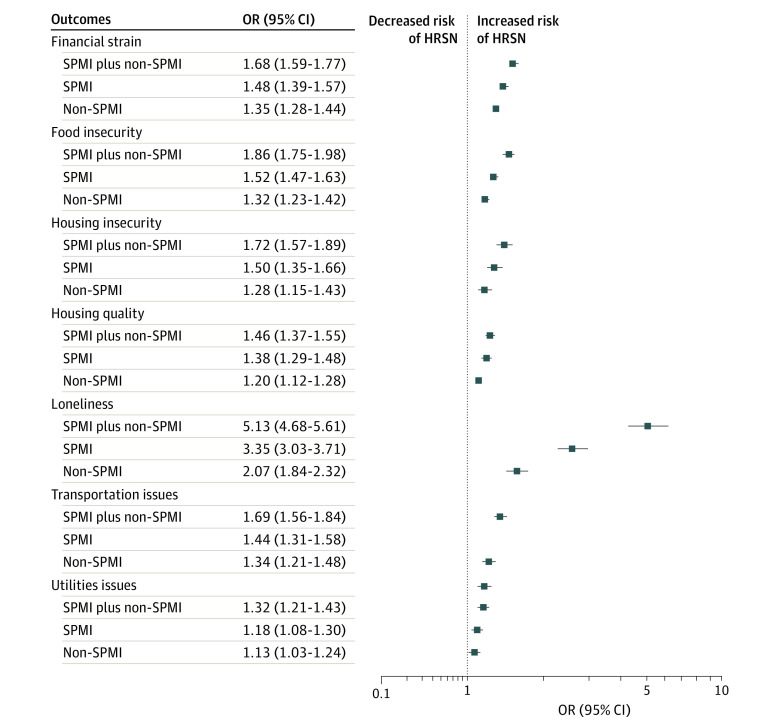
Adjusted Models of the Association Between Mental Illness and the Risk of Specific Health-Related Social Needs (HRSNs) Risk compared with individuals without mental illness. Health-related social needs were self-reported. OR indicates odds ratio; and SPMI, serious persistent mental illness.

The results of the adjusted logistic regression analysis of the association between mental illness in the cohorts with SPMI, non-SPMI, and SPMI plus non-SPMI and specific HRSNs (assessed by plotting the odds ratios [ORs] and their associated 95% CIs) relative to the cohort without mental illness are shown in Figure 2. Each of the 7 models was adjusted for demographic, clinical, and other covariates.

Across all specific HRSNs, the odds of experiencing the respective HRSN increased when compared with the cohort without mental illness in the following cohort order: non-SPMI, SPMI, and SPMI plus non-SPMI. For example, compared with individuals with no mental illness, those with non-SPMI had 35% higher odds of financial strain (OR, 1.35; 95% CI, 1.28-1.44; *P* < .001), those with SPMI had 48% higher odds (OR, 1.48; 95% CI, 1.39-1.57; *P* < .001), and those with SPMI plus non-SPMI had 68% higher odds (OR, 1.68; 95% CI, 1.59-1.77; *P* < .001). The HRSN with the largest risk differences among the study cohorts was severe loneliness; compared with the cohort without mental illness, the non-SPMI cohort had 2.07 times higher odds (95% CI, 1.84-2.32; *P* < .001), the SPMI cohort had 3.35 times higher odds (95% CI, 3.03-3.71; *P* < .001), and the SPMI plus non-SPMI cohort had 5.13 times higher odds (95% CI, 4.68-5.61; *P* < .001) of severe loneliness.

## Discussion

In this large national cohort study of Medicare Advantage enrollees, approximately 4 of 10 individuals (38.6%) had at least 1 mental illness diagnosis in the past year, 5 of 10 (54.0%) had an HRSN, and 1 of 4 (25.3%) had both mental illness and an HRSN. The association of mental illness with the presence of HRSNs was most substantial for those with SPMI plus non-SPMI than for those with only non-SPMI or SPMI. Although the risk of severe loneliness was most noticeable in the cohorts with mental illness, food insecurity, transportation problems, housing instability, housing quality, financial strain, and utility affordability were also more likely in all 3 of the cohorts with mental illness vs the cohort without mental illness. These significant and, in some cases, large incremental increases in the odds of having an HRSN among those with mental illness highlights the need for more upstream targeted interventions to address basic social needs, such as safe housing and food security, for older adults with mental illness, one of the most vulnerable populations.

These findings were generally consistent with those of previous studies. For example, previous research has found that those with mental illness are more likely to have HRSNs, although those studies focused on a subset of social needs^15,42^ or were conducted in international settings.^43^ However, the results of the current study went beyond the commonly reported association of mental illness with HRSNs to suggest that severity of diagnosis and the presence of multimorbidity may be associated with the risk of experiencing HRSNs. The consistent pattern of the SPMI plus non-SPMI cohort being at the highest risk of HRSNs, followed in order by the cohorts with SPMI, non-SPMI, and no mental illness, suggests that patients with a diagnosis of bipolar disorder, major depressive disorder, or schizophrenia may be more vulnerable to multiple social needs. This finding may be partially explained by the fact that the severe impairment associated with SPMI may directly hinder one’s ability to obtain or sustain employment, housing, and personal relationships. Alternatively, the stigmatization of mental illness could also play an indirect role in some HRSNs, such as severe loneliness. These large incremental increases in the odds of experiencing severe loneliness with increasing severity of mental illness diagnosis highlight the association between mental illness burden and loneliness. Severe loneliness has the potential to produce adverse consequences for mental and physical health, often leading to worse health-related quality of life.^44^ The importance of screening for HRSNs in patients with mental illness cannot be understated; the combination of mental illness and HRSNs may be associated with accumulated stress and increases in the likelihood of recurring mental illness exacerbations. It is important to recognize that HRSN screening may not be an approach that is suitable for all patients. Patients with SPMI may need to receive screening on a more frequent basis and may require more intensive remediation of their respective HRSNs. Addressing these upstream social needs will be important to attaining health and wellness for this population.

The CMS developed the AHC model, which provides funding for health care organizations to screen for HRSNs and develop the infrastructure needed to connect individuals with needs to community resources.^45^ Recipients can also use funding to develop networks and enhance coordination across community-based organizations. Through these community coalitions, multiple factors associated with mental health, such as housing, community violence, and education, can be addressed.^46,47^ In response, health care organizations are transforming to better coordinate care by broadening care teams, creating linkages to community organizations, and moving resources out of the clinic and into the community.^48^ Some have succeeded in improving outcomes and reducing costs, but these models require additional training and coordination among payers, practices, hospitals, and public health agencies.^38,49^ The need to refine, implement, and scale new models of delivering public health, mental health, and primary care has only increased during the COVID-19 pandemic.^50,51^

### Public Health Implications

Although true integration of public health, mental health, and primary care remains a goal, value-based care payment models have led to important changes in the delivery of health care.^10,52^ By sharing data, reducing administrative barriers, and realigning financial incentives, these Medicare Advantage programs have reimagined how care is delivered for a wide range of patients with high-cost conditions, including beneficiaries with mental illness.^53^ The findings of this study also have implications for the payment and measurement of care. Given the importance of quality measures in value-based payment arrangements, organizations have called for measures to be adjusted to account for social risk factors and have released reports describing the rationale behind and the methods needed to make such adjustments.^54,55^ These calls have come in response to recognition that individuals with social risk factors have worse outcomes on quality measures and that clinicians caring for these patients have worse performance.^54^ Although this study did not assess the association between HRSNs and adverse patient outcomes or clinician performance, examining whether and to what extent HRSNs are associated with quality of care could guide improvements for patients with mental illness.

### Limitations

This study has several limitations. First, we only included beneficiaries participating in Medicare Advantage from 1 payer; thus, our findings may not be generalizable to those with other Medicare Advantage plans, those with fee-for-service Medicare plans, younger individuals, or those with other types of insurance. Second, our results are subject to recall bias given the nature of survey research as well as the symptoms associated with certain psychiatric disorders.^56,57^ Third, we used administrative claims data to obtain participant demographic and clinical characteristics, which are subject to misclassification and missing data. Fourth, we used a cross-sectional survey that does not allow for the determination of causal pathways between mental illness and HRSNs. Fifth, the use of *ICD-10-CM* codes and the criteria outlined in the Milliman research report^37^ to categorize participants into study cohorts served as a proxy for mental illness severity. However, we did not measure actual impairment at the time of survey administration; future studies could address this limitation by incorporating the World Health Organization Disability Assessment Schedule 2.0.^58^

## Conclusions

The findings of this cohort study highlight the association between mental illness and HRSNs. Although value-based payment models have enhanced the coordination of care, more could be done to ensure the health care system comprehensively and simultaneously addresses physical, mental, and social needs.
